# Effects of Water and Nitrogen Control on the Growth Physiology, Yields, and Economic Benefits of *Lycium barbarum* Plants in a *Lycium barbarum* + Alfalfa System

**DOI:** 10.3390/plants13081095

**Published:** 2024-04-13

**Authors:** Chen Wang, Guangping Qi, Yanlin Ma, Minhua Yin, Jinghai Wang, Yanxia Kang, Qiong Jia, Yalin Gao, Rongrong Tian, Rong Zhang, Qiang Lu, Feng Xiao

**Affiliations:** College of Water Conservancy and Hydropower Engineering, Gansu Agricultural University, Lanzhou 730070, China; wangc@st.gsau.edu.cn (C.W.); mayl@gsau.edu.cn (Y.M.); yinmh@gsau.edu.cn (M.Y.); wangjh@gsau.edu.cn (J.W.); yanxiakang@gsau.edu.cn (Y.K.); jiaq@gsau.edu.cn (Q.J.); gylin@st.gsau.edu.cn (Y.G.); tianrr@st.gsau.edu.cn (R.T.); zhangrong@st.gsau.edu.cn (R.Z.); luq@st.gsau.edu.cn (Q.L.); xiaof@gsau.edu.cn (F.X.)

**Keywords:** *Lycium barbarum* + Alfalfa system, water and nitrogen control, yield, economic benefits, regression analysis

## Abstract

In the production of economic forests, there are common issues such as excessive application of water and fertilizer, redundant plant growth, and low economic benefits. Reasonable water and fertilizer management can not only help address these problems but also improve the absorption and use efficiency of water and fertilizer resources by plants, promoting the green and efficient development of the fruit and forestry industry. In order to explore a suitable water and nitrogen management mode for *Lycium barbarum*, field experiments were conducted in this study from 2021 to 2022. Specifically, four irrigation modes (according to the proportion ratio of soil moisture content to field moisture capacity *θ_f_*, 45–55% *θ_f_* (W1, severe water deficiency), 55–65% *θ_f_* (W2, moderate water deficiency), 65–75% *θ_f_* (W3, mild water deficiency), and 75–85% *θ_f_* (W4, sufficient irrigation)) and four nitrogen application levels (0 kg·ha^−1^ (N0, no nitrogen application), 150 kg·ha^−1^ (N1, low nitrogen application level), 300 kg·ha^−1^ (N2, medium nitrogen application level), and 450 kg·ha^−1^ (N3, high nitrogen application level)) were set up to analyze the influences of water and nitrogen control on the plant height, stem diameter, chlorophyll content, photosynthetic characteristics and yield, and economic benefits of *Lycium barbarum* in the *Lycium barbarum* + Alfalfa system. The study results show that the plant height and stem diameter increment of *Lycium barbarum* increase with the irrigation amount, increasing first and then decreasing with the increase in the nitrogen application level. Meanwhile, the chlorophyll contents in *Lycium barbarum* continuously increase throughout their growth periods, with *Lycium barbarum* treated with W4N2 during all growth periods presenting the highest contents of chlorophyll. In a *Lycium barbarum* + Alfalfa system, the daily variation curve of the *Lycium barbarum* net photosynthetic rate presents a unimodal pattern, with maximum values of the daily average net photosynthetic rate and daily carboxylation rate appearing among W4N2-treated plants (19.56 μmol·m^−2^·s^−1^ and 157.06 mmol·m^−2^·s^−1^). Meanwhile, the transpiration rates of *Lycium barbarum* plants continuously decrease with the increased degree of water deficiency and decreased nitrogen application level. W1N2-treated plants exhibit the highest leaf daily average water use efficiency (3.31 μmol·s^−1^), presenting an increase of 0.50–10.47% in efficiency compared with plants under other treatments. The coupling of water and nitrogen has significantly improved the yields and economic benefits of *Lycium barbarum* plants, with W4N2-treated and W3N2-treated plants presenting the highest dried fruit yield (2623.07 kg·ha^−1^) and net income (50,700 CNY·ha^−1^), respectively. Furthermore, compared with other treatment methods, these two treatment methods (W4N2 and W3N2) exhibit increases of 4.04–84.08% and 3.89–123.35% in dried fruit yield and net income indexes, respectively. Regression analysis shows that, in a *Lycium barbarum* + Alfalfa system, both high yields and economic benefits of *Lycium barbarum* plants can be achieved using an irrigation amount of 4367.33–4415.07 m^3^·ha^−1^ and a nitrogen application level of 339.80–367.35 kg·ha^−1^. This study can provide a reference for improving the productivity of *Lycium barbarum* plants and achieving a rational supply of water and nitrogen in *Lyciun barbarum* + Alfalfa systems in the Yellow River Irrigation Area of Gansu, China, and other similar ecological areas.

## 1. Introduction

*Lycium barbarum* L. is a deciduous shrub species belonging to the Solanaceae family. First recorded in the *Shennong Bencaojing*, this plant presents not only such pharmacological effects as improving immunity, aging resistance, tumor resistance, and neuroprotection [1] but also strong ecological adaptability to drought, salinity, and alkalinity, making it an excellent tree species for wind protection and sand fixation in desert areas [2,3]. *Lycium barbarum* is cultivated in South America, Australia, Eurasia, and other regions. China is the largest producer of *Lycium barbarum* in the world, accounting for over 80% of the global cultivation area of *Lycium barbarum* [4,5]. In recent years, the production of *Lycium barbarum* plants in the middle and upper reaches of the Yellow River in Ningxia, Gansu, Qinghai, and Inner Mongolia in China has increased from 190,000 tons to around 420,000 tons, making this plant species a characteristic crop promoting the economic growth in these areas [6,7,8]. However, in production, farmers generally adhere to the concept of “sufficient irrigation and fertilization leading to high yields”, which not only results in a serious waste of water and fertilizer resources and pollution of soil environments but also brings about nutrient and growth redundancy of *Lycium barbarum* tree crowns and increased planting costs, thus severely hindering the green and ecological development of the Chinese *Lycium barbarum* industry [9,10]. In the strategic context of implementing ecological protection and high-quality development in the Yellow River Basin in China, how to ensure the green and sustainable development of the *Lycium barbarum* industry in the upper and middle reaches of the Yellow River has become an important issue that needs to be urgently addressed [11,12].

Water and nitrogen are key factors that affect the growth and development of crops. Water content is not only an important component of plants but also an indispensable medium for plant metabolism [13,14]. Studies have shown that the annual net growth amounts of *Lycium barbarum*’s plant height and stem diameter increase with the irrigation amount. However, when the irrigation amount reaches a certain level, the increased amplitude of stem diameter starts to decrease [15]. Under an irrigation amount of 4500 m^3^·ha^−1^, young *Lycium barbarum* trees present the optimal leaf morphology, structures, and functions, with the strongest photosynthesis [16]. A certain degree of water deficiency can lead to increased yields of *Lycium barbarum* plants. However, under a severe water deficiency, their yields will show a decreasing trend [17]. Nitrogen is an important component of plant proteins, nucleic acids, enzymes, and hormones and is referred to as “the element of life” [18,19]. Studies have indicated that the application of nitrogen fertilizer can significantly promote the plant height, stem diameter, crown size, and leaf area of *Lycium ruthenicum* [20]. A nitrogen application amount of 675 kg·ha^−1^ can not only facilitate the root system growth and development of *Lycium barbarum* plants and construct the good morphology of root systems but also promote the photosynthesis of plants and improve their fruit quality and production benefits [21]. With an increase in the amount of nitrogen applied, *Lycium barbarum* plant yields present a trend of increasing first and then decreasing. Meanwhile, an increased amount of nitrogen application can increase the nitrogen absorption amounts of *Lycium barbarum* plants and fruits [22]. Coordinated regulation of water and nitrogen can promote the growth and development of crops and increase the accumulation of photosynthetic products through the coupling and complementary effect of water and nitrogen [23]. Research shows that water and nutrient contents required by crops during their peak growth periods in an intercropping system can be supplied through irrigation and nitrogen application [24]. Irrigation and nitrogen application have a significant influence on the plant height, stem diameter, and leaf area indexes of soybeans in intercropping systems [25]. Net photosynthetic rates and transpiration rates of crops in an apple and soybean system both reach their maximum levels under a moderate fertilization level (92 kg·ha^−1^) combined with a mild irrigation deficiency level (80% *θ_f_*). In addition, the water use efficiency and yields of soybeans present a pattern of increasing first and then decreasing with the increases in amounts of irrigation and nitrogen application [26,27]. In an apple + corn system, the growth and yields of corn increase with the irrigation amount, presenting a negative correlation with the amount of nitrogen application. Under a sufficient irrigation treatment (85% *θ_f_*), the net photosynthetic rates and leaf water use efficiency of crops reach their maximum levels, and a low nitrogen application level (298 kg·ha^−1^) is conducive to improving the photosynthetic efficiency of the system [28].

In summary, current studies on *Lycium barbarum* have mostly focused on a single factor of irrigation or nitrogen application [15,16,17,18,19,20,21,22], and most studies exploring the influence of water and nitrogen coupling on intercropping systems have focused on such crops as apples, soybeans, and corn [24,25,26,27,28], with fewer studies dealing with perennial *Lycium barbarum* plants in intercropping systems. The practice of intercropping not only enhances the efficiency of water, fertilizer, gas, and heat resource utilization but also contributes to soil structure improvement, disease and pest control, as well as biodiversity enhancement. These benefits are crucial for strengthening and stabilizing agricultural ecosystems. The current combined planting of *Lycium barbarum* and Alfalfa plants in the Yellow River Irrigation Area in Gansu, China is a planting model that combines economic, ecological, and social benefits. This model has transcended the single production method of *Lycium barbarum* or Alfalfa crops, presenting a huge potential for such aspects as improving soil salinization, controlling soil erosion, increasing biological nitrogen fixation, and enhancing land productivity [29,30,31,32,33]. Therefore, this study focuses on the *Lycium barbarum* crops in *Lycium barbarum* + Alfalfa systems as a research object, with the following aims: (1) systematically analyze the influences of the water and nitrogen control on the growth physiology, yields, and economic benefits of *Lycium barbarum* crops; (2) explore the appropriate range of water and nitrogen supply through regression analysis to provide theoretical evidence for the green and high-quality development of *Lycium barbarum* plants in *Lycium barbarum* + Alfalfa systems in the Yellow River Irrigation Area of Gansu, China and other similar ecological areas.

## 2. Results

### 2.1. Impact of Water and Nitrogen Control on the Growth of Lycium barbarum Plants in a Lycium barbarium + Alfalfa System

#### 2.1.1. Plant Height Growth Amount

**Figure 1:**
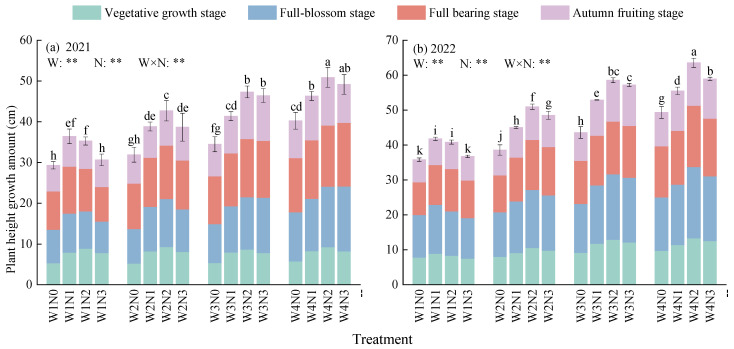
Effect of water and nitrogen treatment on the plant height growth amount of *Lycium barbarum*. W1, W2, W3, and W4 represent the irrigation modes of severe deficit, moderate deficit, mild deficit, and full irrigation, respectively. N0, N1, N2, and N3 indicate no nitrogen, low nitrogen, medium nitrogen, and high nitrogen application levels, respectively. Different lowercase letters indicated significant difference under different treatments (*p* < 0.05). W is water mode, N is nitrogen application level, and W × N is their interaction. ** indicates that there is a very significant difference (*p* < 0.01).

The plant height growth amount of *Lycium barbarum* is significantly impacted by the irrigation and nitrogen application amounts and the interactive effect between water and nitrogen (*p* < 0.01, Figure 1). Throughout the growth period of *Lycium barbarum*, its plant height growth amount increases with the irrigation amount, increasing first and then decreasing with the increase in the nitrogen application amount. Under the irrigation modes of W1, W2, W3, and W4, the average plant height growth amounts at different nitrogen application levels fell into ranges of 32.91–46.63 cm and 38.79–56.83 cm in 2021 and 2022, respectively. Meanwhile, in 2021, the above-mentioned growth amounts under the W4 mode were 10.05%, 22.67%, and 41.72% higher than those under the W3, W2, and W1 modes, respectively. In addition, these figures increased to 7.11%, 24.23%, and 46.51% in 2022. Similarly, at the nitrogen application levels of N1, N2, N3, and N4, the average plant height growth amounts under different irrigation modes fell into ranges of 33.98–44.02 cm in 2021 and 41.82–53.47 cm in 2022. In addition, in 2021, the above-mentioned growth amounts under the N2 level were 6.81%, 8.21%, and 29.57% higher than those under the N3, N1, and N0 levels, respectively. Furthermore, these figures increased to 6.21%, 9.59%, and 27.86% in 2022. Among different types of water and nitrogen treatment, all W4N2-treated plants presented maximum plant height growth amounts throughout their growth periods in 2021 and 2022 (50.87 cm and 63.54 cm), presenting increases of 3.52–73.56% and 7.82–77.54% over those growth amounts of plants with other treatment types in 2021 and 2022, respectively. This indicates that a combination of sufficient irrigation and moderate nitrogen application can better facilitate the plant height growth of *Lycium barbarum.*

#### 2.1.2. Stem Diameter Growth Amount

**Figure 2:**
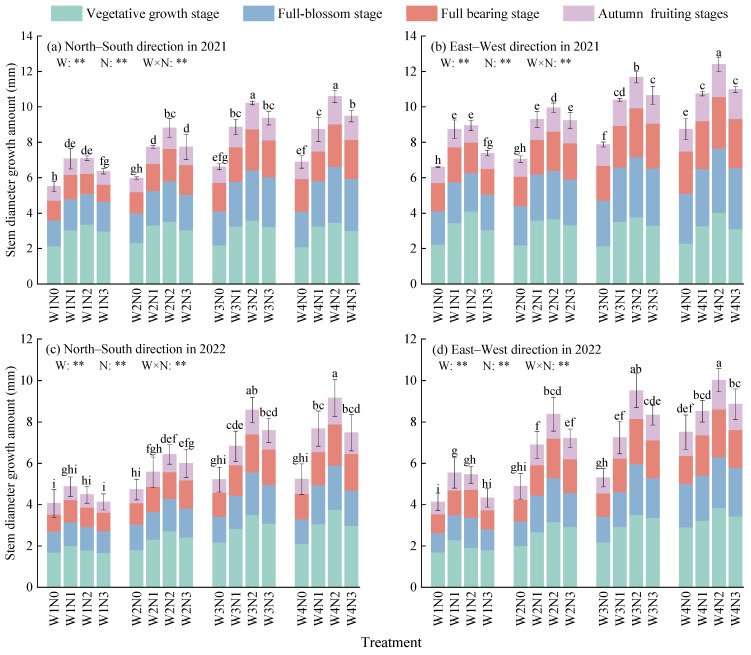
Effect of water and nitrogen treatment on the stem diameter growth amount of *Lycium barbarum*. W1, W2, W3, and W4 represent the irrigation modes of severe deficit, moderate deficit, mild deficit, and full irrigation, respectively. N0, N1, N2, and N3 indicate no nitrogen, low nitrogen, medium nitrogen, and high nitrogen application levels, respectively. Different lowercase letters indicated significant difference under different treatments (*p* < 0.05). W is water mode, N is nitrogen application level, and W × N is their interaction. ** indicates that there was a very significant difference (*p* < 0.01).

The stem diameter growth amount of *Lycium barbarum* during its entire growth period is significantly affected by irrigation, nitrogen application, and the interactive effect between water and nitrogen (*p* < 0.01, Figure 2). Ranges of stem diameter growth amounts (average values along the south–north and east–west directions) of *Lycium barbarum* plants under different types of water and nitrogen treatment during their various growth periods were 1.74 mm, 1.71 mm, 1.44 mm, and 0.89 mm in 2021, and 2.13 mm, 1.31 mm, 1.29 mm, and 0.79 mm in 2022. All plant growth periods are listed as follows in the decreasing order of value differences: the vegetative growth period, the full bloom period, the full bearing period, and the autumn fruiting period. Water and nitrogen have the most significant effect on the stem diameter growth amounts of *Lycium barbarum* plants during the vegetative growth period. Under the same nitrogen application level, the stem diameter growth amounts of *Lycium barbarum* plants increase with the amount of irrigation. In addition, compared with those growth amounts of plants treated with other irrigation modes, the stem diameter growth amounts of W4-mode plants were increased by 3.77–86.12% in 2021 and 2.70–93.26% in 2022. Under the same mode of irrigation, the stem diameter growth amounts of plants increase first and then decrease with the increase in the nitrogen application level. Meanwhile, N2–level plants present the highest growth amounts of stem diameters, which were 7.65–42.86% and 12.20–72.05% higher than those growth amounts of plants with other nitrogen application levels in 2021 and 2022, respectively. Among all treated plants, W4N2–treated plants present the highest stem diameter growth amounts throughout their growth periods, which were 28.46% and 7.97% higher than those of W4N0–treated and W4N3–treated plants in 2021, respectively (30.57% and 17.71% in 2022).

### 2.2. Influence of Water and Nitrogen Control on the Physiology of Lycium barbarum Plants in a Lycium barbarum + Alfalfa System

#### 2.2.1. Chlorophyll Content(SPAD)

**Figure 3:**
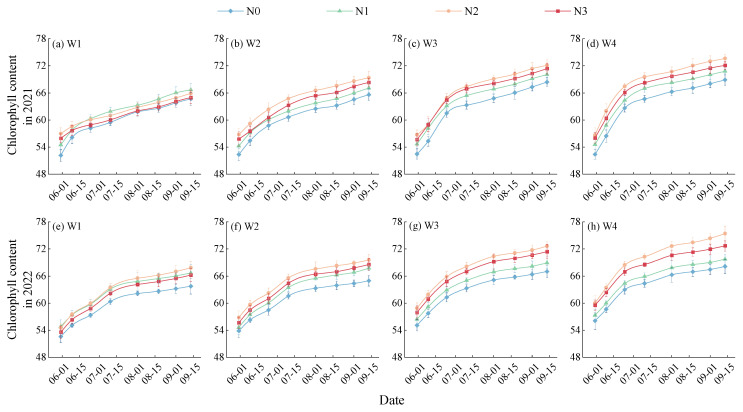
Effect of water and nitrogen treatment on the chlorophyll content (SPAD) of *Lycium barbarum*. W1, W2, W3, and W4 represent the irrigation modes of severe deficit, moderate deficit, mild deficit, and full irrigation, respectively. N0, N1, N2, and N3 indicate no nitrogen, low nitrogen, medium nitrogen, and high nitrogen application levels, respectively.

Leaf SPADs of *Lycium barbarum* plants continuously increase with the progression of their growth periods, with differences among differently-treated plants becoming more significant (Figure 3). SPADs of *Lycium barbarum* plants increase rapidly during their vegetative growth and full-blossom periods; during their full bearing and autumn fruiting periods, the increases in their SPADs slow down. Under a same irrigation mode, leaf SPADs of *Lycium barbarum* increase first and then decrease with the increase in the nitrogen application amount, with N2–level plants presenting the highest SPAD levels. Under the same level of nitrogen application, SPADs of all treated plants consistently increase with the irrigation amount. Among all *Lycium barbarum* plants treated with different water and nitrogen control modes, W4N2–treated plants present the highest leaf SPAD values during the vegetative growth, full–blossom, full bearing, and autumn fruiting periods, which are 2.19–12.80%, 2.20–16.69%, 2.52–16.18%, and 2.91–16.06% higher than those two-year average values of plants under other treatment modes during the same periods, respectively.

#### 2.2.2. Photosynthetic Characteristics

(1) Net Photosynthetic Rate (*Pn*)

**Figure 4:**
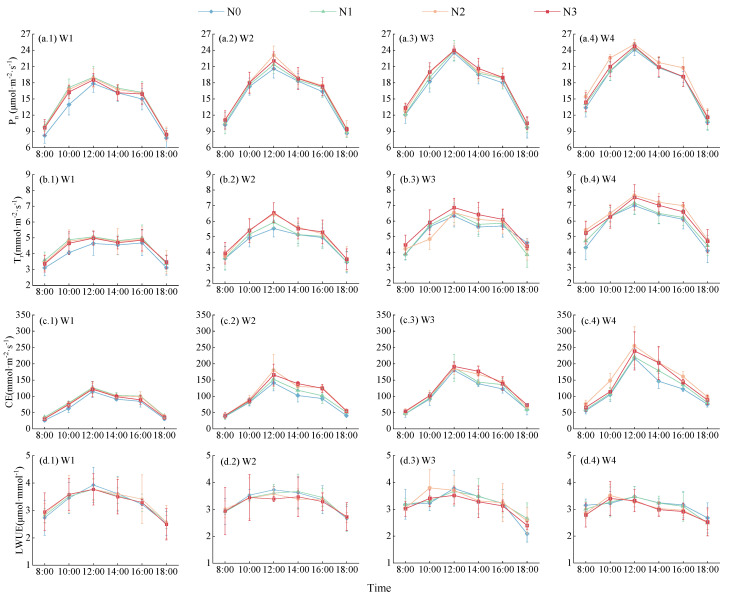
Effect of water and nitrogen treatment on the photosynthetic characteristics of *Lycium barbarum*. W1, W2, W3, and W4 represent the irrigation modes of severe deficit, moderate deficit, mild deficit, and full irrigation, respectively. N0, N1, N2, and N3 indicated no nitrogen, low nitrogen, medium nitrogen, and high nitrogen application levels, respectively. (**a.1**–**a.4**) represent net photosynthetic rates under W1, W2, W3, and W4 irrigation patterns. (**b.1**–**b.4**) represent transpiration rates under W1, W2, W3, and W4 irrigation patterns. (**c.1**–**c.4**) represent carboxylation rates under W1, W2, W3, and W4 irrigation patterns. (**d.1**–**d.4**) represent leaf instantaneous water use efficiency under W1, W2, W3, and W4 irrigation patterns.

Daily variation curves of *Lycium barbarum* leaf *Pn* under different water and nitrogen treatment modes present a unimodal shape, with their peak values all appearing at 12:00 (Figure 4a). Under a same nitrogen application level, all *Lycium barbarum Pn* values at different moments increase with the amount of irrigation, with W4–mode plants presenting 3.29–32.58% higher *Pn* peak values and 6.91–32.00% higher daily average *Pn* values than those of plants treated with other modes. Under a same irrigation mode, all *Lycium barbarum Pn* values at different moments increase first and then decrease with the increase in the nitrogen application level, with N2–level plants presenting 1.82–5.75% higher *Pn* peak values and 1.82–7.58% higher daily average *Pn* values than those of plants under other nitrogen application levels. Among all treated plants, W4N2–treated plants present the highest *Pn* peak value and daily average *Pn* value, which are 25.12 μmol·m^−2^·s^−1^ and 19.56 μmol·m^−2^·s^−1^, respectively.

(2) Transpiration Rate (*T_r_*)

The daily variation leaf *Tr* curve of W1–mode *Lycium barbarum* plants presents a bimodal shape, while the curves of plants with other treatment modes present a unimodal shape (Figure 4b). Peak *Tr* values (maximum peak values) of plants under different water and nitrogen treatment modes all appear at 12:00. Under the W1 and W4 treatment modes, *Tr* values increase first and then decrease with the increase in the nitrogen application level; under the W1 mode, N1–level plants present a daily average *Tr* value 1.37–10.60% higher than those of plants treated with other nitrogen application levels. Likewise, under the W4 treatment mode, N2–level plants present a daily average *Tr* value 3.29–12.74% higher than those of plants treated with other application levels. Under the W2 and W3 treatment modes, *Tr* values consistently increase with the nitrogen application level. At the N3 level, W2–mode and W3–mode plants present 0.80–10.07% and 7.31–7.75% higher daily average *Tr* values than those of plants under other nitrogen application levels. Under all nitrogen application levels, *Tr* values continuously increase with the irrigation amount, with W4–mode plants presenting a *Tr* value 12.40–40.98% higher than those of plants under other treatment modes. W4N2–treated plants present the highest daily average *Tr* value (6.43 mmol·m^−2^·s^−1^), which is 3.29–59.86% higher than those values of plants under other treatment modes.

(3) Carboxylation Efficiency (*CE*)

The carboxylation efficiency of a crop refers to the speed at which the crop undergoes photosynthesis, absorbing carbon dioxide from the atmosphere and converting it into organic matter. This process directly impacts both the growth rate and final yield of crops. Under the same nitrogen application level, the daily average leaf *CE* of *Lycium barbarum* consistently increases with the irrigation amount, with an average value range of 94.59–114.86 mmol·m^−2^·s^−1^ among all four irrigation modes. Meanwhile, W4–mode plants present a daily average leaf *CE* value 81.73%, 44.93%, and 19.56% higher than those of W1–mode, W2–mode, and W3–mode plants, respectively. Under a same irrigation mode, the daily average *CE* of *Lycium barbarum* plants increases first and then decreases with the increase in the nitrogen application amount, with an average value of 75.79–137.74 mmol·m^−2^·s^−1^ among all four nitrogen application levels. In addition, N2–level plants present a daily average *CE* value 21.43%, 10.89%, and 0.04% higher than those of N0–level, N1–level, and N3–level plants, respectively. Furthermore, W4N2–treated and W1N0–treated *Lycium barbarum* plants present the highest daily average *CE* value of 157.06 mmol·m^−2^·s^−1^ and the lowest daily average *CE* value of 68.43 mmol·m^−2^·s^−1^, respectively (Figure 4c).

(4) Leaf Instantaneous Water Use Efficiency (*LWUE*)

*Lycium barbarum LWUE* presents an increasing and then decreasing pattern with the increase in the nitrogen application amount and a continuously decreasing pattern with the increase in the irrigation amount (Figure 4d). Under the W1, W2, W3, and W4 modes, maximum peak values of *LWUE* are presented among W1N0–treated (3.93 μmol·s^−1^), W2N0–treated (3.73 μmol·s^−1^), W3N2–treated (3.80 μmol·s^−1^), and W4N2–treated (3.51 μmol·s^−1^) plants, with peak values occurring at the moments of 12:00, 12:00, 10:00, and 10:00, respectively. Meanwhile, the daily average *LWUE* value of W1–mode *Lycium barbarum* plants is 0.14–6.51% higher than those of plants treated with other modes. Under the N0, N1, N2, and N3 treatment levels, maximum peak values of *LWUE* are presented among W1N0–treated (3.93 μmol·s^−1^), W1N1–treated (3.77 μmol·s^−1^), W3N2–treated (3.80 μmol·s^−1^), and W1N3–treated (3.77 μmol·s^−1^) plants, with peak values occurring at the moments of 12:00, 12:00, 10:00, and 12:00, respectively. In addition, N2–level *Lycium barbarum* plants present the highest value of daily average *LWUE*. Under different water and nitrogen treatment modes, W1N2–treated *Lycium barbarum* plants present the highest daily average *LWUE* value, which is 0.50–10.47% higher than those values of plants under other treatment modes.

### 2.3. Influences of Water and Nitrogen Control on the Yields and Economic Benefits of Lycium barbarum Crops in a Lycium barbarum + Alfalfa System

#### 2.3.1. Yields and Economic Benefits 

Irrigation, nitrogen application, and their interaction can significantly affect the dried-fruit yields and economic benefits of *Lycium barbarum* (*p* < 0.01, Table 1). All irrigation modes are listed as follows in descending order of *Lycium barbarum* dried-fruit yields: W4 > W3 > W2 > W1; W4–mode plants present a dried-fruit yield 2.47–47.74% higher than those of plants treated with other irrigation modes. Likewise, these irrigation models are listed as follows in descending orders of *Lycium barbarum* net profits and cost–benefit ratios, respectively: W3 > W4 > W2 > W1 and W3 > W2 > W4 > W1. W3–mode plants present a 3.96–55.67% higher net profit and 2.39–8.34% higher cost–benefit ratio than those of plants with other treatment modes. All nitrogen application levels are listed as follows in descending order of dried-fruit yields/net profits of *Lycium barbarum* crops: N2 > N3 > N1 > N0; N2–level plants present a 7.19–26.08% higher yield and a 3.88–46.79% higher net profit than those of plants with other nitrogen application levels. Likewise, these nitrogen application levels are listed as follows in descending order of cost–benefit ratios: N3 > N2 > N1 > N0; N3–level plants present a ratio 3.28–19.84% higher than those of plants with other nitrogen application levels. Among all treated plants, W4N2–treated *Lycium barbarum* plants present the highest dried-fruit yield of 2623.07 kg·ha^−1^, W3N2–treated plants present the highest net profit of 50,700 CNY·ha^−1^, and W3N3–treated plants present the highest cost–benefit ratio of 2.34.

#### 2.3.2. Regression Analysis of the Relationship between Water and Nitrogen Supply and Plant Yields/Net Profits 

**Figure 5:**
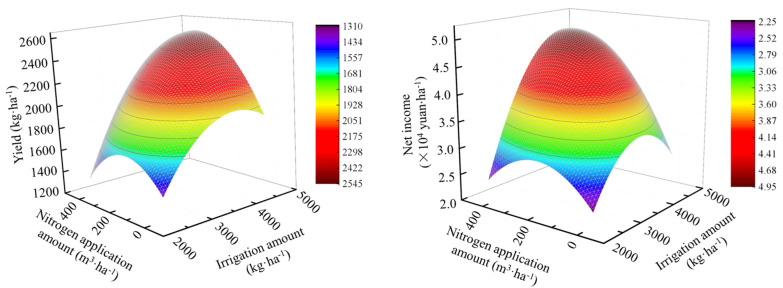
Regression equation of water and nitrogen dosage and yield and net income of *Lycium barbarum*.

Regression equations (Equations (1) and (2)) and regression model (Figure 5) of *Lycium barbarum* dried-fruit yields and net profits with the application amounts of water and nitrogen show that under two optimal combinations of irrigation and nitrogen application amounts (4606.01 m^3^·ha^−1^/339.80 kg·ha^−1^ and 4415.07 m^3^·ha^−1^/394.90 kg·ha^−1^), maximum yields, and maximum net profits of *Lycium barbarum* crops can be achieved, respectively. These two combinations of water and nitrogen application amounts required for achieving the maximum yields and the maximum net profits are not consistent with each other. Therefore, with the aim of achieving 95% of maximum yield and net profit values, a regression analysis was performed, with the result that a combination of an irrigation amount range of 4367.33–4415.07 m^3^·ha^−1^ and a nitrogen application amount range of 339.80–367.35 kg·ha^−1^ can be used as an optimal water and nitrogen control method to achieve high yields and economic benefits of *Lycium barbarum* crops.

(1) $$Y=-2.03301\times 10^{-4}W^{2}-5.58\times 10^{-3}N^{2}+6.75491\times 10^{-4}WN+1.65093W+0.70144N-1401.9861\;(R^{2}=0.963\;**)$$

(2) $$NP=-5.90262\times 10^{-7}W^{2}-1.2608\times 10^{-5}N^{2}+2.05253\times 10^{-6}WN+4.39\;\times \;10^{-3}W+8.77426\times 10^{-4}N-4.90808\;(R^{2}=0.963\;**)$$

## 3. Discussion

### 3.1. Impact of Water and Nitrogen Control on the Growth of Lycium barbarum Plants

Both excessive and insufficient supply of water and nitrogen can inhibit the growth of crops [34,35], while the scientific and rational application of water and nitrogen can not only promote the growth and development of crops but also increase their yields [36,37,38]. Mofokeng et al. [39] studied the growth responses of geraniums to water and nitrogen in Pretoria, South Africa and found that water stress can significantly reduce the single-plant leaf numbers, plant heights, and fresh-root yields of geraniums, while a moderate nitrogen application level can increase the leaf area index of this plant species and promote their growth. Similarly, He et al. [40] studied the growth of poplar trees in Shandong, China and discovered that this tree species presents a positive correlation between its growth and irrigation amount, while a high nitrogen application level presents no promotion effect on its growth. This study also demonstrates that both the plant height and stem-diameter growth amounts of *Lycium barbarum* plants consistently increase with the irrigation amount and present an increasing and then decreasing pattern with the increase in the nitrogen application level, and W4N2–treated plants present maximum values of these two growth amounts. A possible reason is that sufficient irrigation combined with moderate nitrogen application can enhance the activities of soil enzymes and microorganisms and promote the conversion and absorption of nutrient substances through root systems [41], thus improving the growth and development of plants [42,43]. However, Liu et al. [44] studied pear trees in northern China and found that under an irrigation lower limit of 65% *θ_f_* and a nitrogen application level of 486 kg·ha^−1^ the treetop length in spring, basal diameters, and relative leaf chlorophyll contents of pear trees are significantly increased. This presents some differences from the study result that *Lycium barbarum* plants treated with sufficient irrigation combined with moderate nitrogen application can achieve maximum growth amounts in their plant heights and stem diameters. Through analysis, one possible reason was obtained that after the intercropping of *Lycium barbarum* plants with Alfalfa plants as one type of leguminous forage grass, through the biological nitrogen fixation effect, Alfalfa plants can not only obtain the nitrogen amounts to meet their own growth needs but also provide some certain amounts of nitrogen for the growth of *Lycium barbarum* crops. However, after the intercropping of Alfalfa plants, increased water consumption in the system leads to increased demand for soil moisture contents. Therefore, increased irrigation can weaken the competition between *Lycium barbarum* and Alfalfa plants for water, thus leading to increased growth rates of *Lycium barbarum* crops.

### 3.2. Influence of Water and Nitrogen Control on the Physiology of Lycium barbarum Plants

Chlorophyll is an important photosynthetic pigment, and only through chlorophyll can photochemical reactions be initiated by light energy [45]. Normal photosynthesis is a prerequisite for crop metabolism and yield formation. However, it is susceptible to changes in external environmental factors such as irrigation levels and nitrogen application amounts [46]. Previous studies have shown that no nitrogen application and severe water deficiency can both significantly reduce the photosynthetic performance of crops [26,47]. In this study, it was found that the *Pn*, *Tr*, and *CE* values of *Lycium barbarum* continuously decrease with the decrease in the irrigation amount, while the *LWUE* value of *Lycium barbarum* continuously increases with the decrease in the irrigation amount. The possible reason could lie in two aspects, as follows: On the one hand, water deficiency leads to decreased leaf chlorophyll contents, increased leaf radical numbers, and over-oxidative photosynthetic pigment membrane systems among *Lycium barbarum* crops, thus increasing the resistance to the synthesis of photosynthetic pigments and ultimately weakening photosynthesis [13,48,49,50]. On the other hand, with the decrease in the irrigation amount, leaf stomatal densities and apertures of *Lycium barbarum* plants gradually decrease, thus further reducing their gas exchange efficiency and altering the coupling relationships among *Pn*, T_r_, and C_i_ [13]. Furthermore, this study concluded that daily average values of *Pn*, *CE*, and *LWUE* of the *Lycium barbarum* plant increase first and then decrease with the increase in the nitrogen application amount, and its *Pn*, *CE*, and *LWUE* reach their maximum levels under the N2 nitrogen application level, which is consistent with the results obtained by Liang et al. [51] in their studies in Xinjiang, China on cotton + mung bean systems. An excessive or insufficient supply of nitrogen could lead to reduced chlorophyll soluble protein levels and activities of photosynthetic enzymes within plants, thus reducing the photosynthetic capacities of leaves [52,53]. An appropriate nitrogen application level can lead to increased contents of chlorophyll and enhanced efficiency of photosynthetic activities [54,55], thus promoting the light energy conversion efficiency of plants and further enhancing the photosynthetic effect of plants [56,57]. In addition, this study shows that *Pn*, *Tr*, *CE*, and *LWUE* values of *Lycium barbarum* plants measured before 12:00 are all significantly higher than those of *Lycium barbarum* plants measured after 12:00. This is possibly due to the fact that favorable field environmental conditions, including temperature, light intensity, and humidity in the morning, are conducive to the photosynthetic conversion of *Lycium barbarum* leaves, accelerating the synthesis of photosynthetic products. Meanwhile, high temperatures and radiation in the afternoon result in imbalanced water contents within plants, decreased activities of photosynthetic enzymes, and slowly recovered stomatal conductance, thus leading to weakened plant photosynthesis [58,59,60].

### 3.3. Influence of Water and Nitrogen Control on the Yields and Economic Benefits of Lycium barbarum Crops

Water and nitrogen coupling can increase crop yields and save costs to a certain extent, and yields and economic benefits are important indicators of high yields and efficiency of crops [61]. This study shows that with the increase in the irrigation amount, the yields of *Lycium barbarum* crops continuously increase, and the net income increases first and then decreases. Meanwhile, with the increase in the amount of nitrogen application, the yields and net income of *Lycium barbarum* crops increase first and then decrease. This is consistent with the results obtained by Ma et al. [34] in their studies on *Lycium barbarum* in Ningxia, China and the results obtained by Wang et al. [62] in their studies on awnless brome in Ningxia, China. Therefore, it can be seen that appropriate water and nitrogen supply is an effective way to enhance crop yields and economic benefits. In their studies on the Loess Plateau in China, Hao et al. [63] applied the TOPSIS method combined with regression analysis and concluded that a water and nitrogen management mode that applies an irrigation amount range of 95–115 mm and a nitrogen application amount range of 470–575 kg ha^−1^ could lead to water and nitrogen conservation, increased yields, and high-quality products in the apple cultivation. Based on AHP and the entropy weight method, Deng et al. [64] conducted a comprehensive assessment study on the combined application of water and nitrogen in *Lycium barbarum* cultivation and found that an optimal comprehensive benefit of *Lycium barbarum* crops can be achieved under an irrigation quota range of 2520–2780 m^3^·ha^−1^ (excluding spring and winter irrigation) and a nitrogen application range of 197–203 kg·ha^−1^. Through regression prediction, in their studies in Gansu, China, Tang et al. [65] concluded that the appropriate irrigation and nitrogen application amount ranges in the cultivation of *Bromus Inermis* brome plants in the Hexi Corridor Region are 546.3–552.5 mm and 136.8–152.3 kg·ha^−1^, respectively. In this study, it has been discovered that, in order to maximize economic benefits, a high level of nitrogen application is required. However, a high level of nitrogen application can not only lead to resource waste but also bring about a series of environmental issues, making it difficult to maximize yields and economic benefits at the same time in crop cultivation. Therefore, through the analysis of *Lycium barbarum* growth physiological indicator and its yield–benefit indicator, representative *Lycium barbarum* dried-fruit yields and net incomes were selected and combined with their water and nitrogen application amounts to establish two binary equations for regression analysis, with the conclusion that the optimal irrigation amount range of 4367.33–4415.07 m^3^·ha^−1^ combined with the optimal nitrogen application range of 339.80–367.35 kg·ha^−1^ can lead to excellent yields and physiological growth of *Lycium barbarum* crops. The results mentioned above were obtained through studies focusing on different research methods and indicators. In addition, these results are significantly influenced by crop, regional, and environmental conditions. Therefore, there are significant differences in rational water and fertilizer management measures that were derived. Optimizing crop water and nitrogen control modes based on local conditions can not only ensure healthy growth and development of crops but also realize high cultivation yields and efficiency.

## 4. Materials and Methods

### 4.1. Description of the Experimental Site

**Figure 6:**
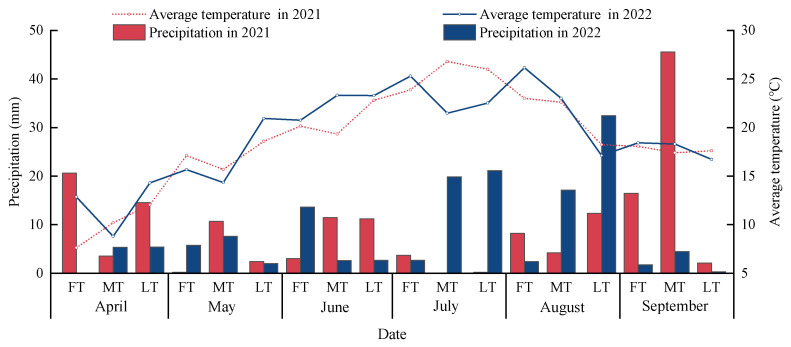
Precipitation and average temperature during the 2021–2022 trial period. FT indicates the first ten days, MT indicates the middle ten days, and LT indicates the last ten days.

Experiments were conducted from March to October in 2021 and 2022 at the irrigation test station of the Jingtaichuan Electric Irrigation Water Source Utilization Uenter in Gansu Province (37°12′59″ N, 104°05′10″ E, with an average altitude of 1562 m). This area is located in the upper and middle reaches of the Yellow River, with a temperate continental arid climate. In this area, there is an average annual precipitation amount of 201.6 mm, an average annual evaporation amount of 2761 mm, and an average annual temperature of 8.6 °C, with an annual frost-free period of 191 d, an annual sunshine duration of 2652 h, and an average annual radiation amount of 6.18 × 10^5^ J·cm^−2^. The soil in the experimental area belongs to a sandy loam type with a bulk density of 1.66 g·cm^−3^, a field water holding capacity of 24.13% (volumetric water content), and a pH value of 8.11. The soil basic nutrient contents within a depth range of 0–60 cm include organic matter (6.09 g·kg^−1^), total nitrogen (1.62 g·kg^−1^), available nitrogen (74.51 mg·kg^−1^), available phosphorus (26.32 mg·kg^−1^), and available potassium (173 mg·kg^−1^). In addition, the average precipitation amounts and temperatures during the two-year experimental period are 170.46 mm and 18.73 °C in 2021, respectively, and 147.29 mm and 19.07 °C in 2022, respectively (Figure 6). 

### 4.2. Experimental Design and Field Management

**Figure 7:**
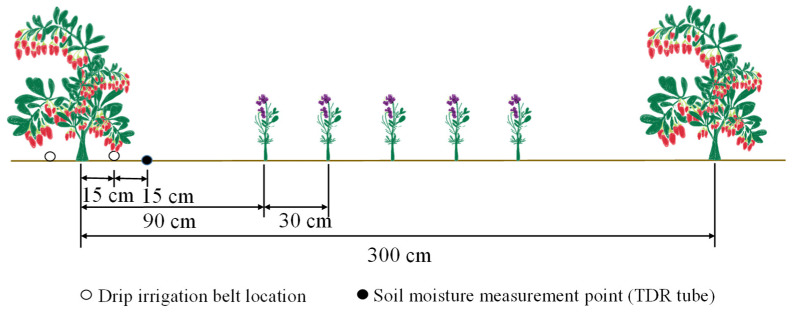
Test plot layout.

With reference to previous studies [33,64] and local farmer management experiences, a completely random combination design was applied in the experiments, with the set-up of two factors: irrigation (irrigation was performed when the soil water content in the planned soil moisture layer dropped to the lower limit of water control) and nitrogen application (urea (with a nitrogen content of 46%) was used as the nitrogen fertilizer, which was applied at a ratio of 6:2:2 during the periods of vegetative growth, full bloom, and full bearing). Four levels were set up in each factor with a total of sixteen treatments, and each treatment was repeated three times (Table 2). The artificial combined cultivation of *Lycium barbarum* and Alfalfa plants was completed in late March 2021, Wate and nitrogen control treatments were started in mid-May 2021 and mid-May 2022. Seedlings of biennial “Ningqi No.7” *Lycium barbarum* were planted with a plant spacing of 1.5 m × 3 m. The “Longdong Alfalfa” variety was applied in this study with a seeding rate of 13 kg·ha^−1^. Meanwhile, Alfalfa seeds were sowed with five sowing rows with a row spacing of 0.3 m arranged at a distance of 0.9 m from *Lycium barbarum* trunks, covering a plot area of 78.75 m^2^. Phosphorus fertilizer (calcium superphosphate, P_2_O_5_, 12%) and potassium fertilizer (powdered potassium, K_2_O, 60%) were used as base fertilizers and applied once during the budding period each year, with an application rate of 130 kg·ha^−1^ (pure amount). Drip irrigation is the method used in the intercropping system, the base fertilizer (phosphate and potassium) is applied by digging holes, and the nitrogen fertilizer is supplied to the plants via an integrated water and fertilizer device (Venturi tube). Drip irrigation was carried out using two drip irrigation belts arranged 0.15 m away from *Lycium barbarum* trunks at the south and north sides (Figure 7). Regulating pools, water pumps, filtration devices, main pipes, branch pipes, and capillary pipes (built-in patch drip irrigation pipes with an inner diameter of 16.0 mm, a drip head spacing of 0.3 m, and a drip flow rate of 2.0 L·h^−1^) were arranged in the field irrigation system. A water meter (with an accuracy of 0.0001 m^3^) was arranged in each experimental plot to control the irrigation amount. Field management measures consistent with local planting habits, including orchard weeding, pruning, and pest and disease prevention and control, were applied in the experiments. Based on the growth pattern of *Lycium barbarum* plants, their growth periods can be divided into five stages as follows [33,64]: the germination stage (from late April to late May), the vegetative growth stage (from late May to early June), the full-blossom stage (from early June to mid-July), the full bearing stage (from mid-July to mid-August) and the autumn fruiting stage (from mid-August to mid-September).

### 4.3. Indicators and Methods for Measurement

#### 4.3.1. Soil Moisture Content

Soil moisture contents in soil layers within the depth ranges of 0–20 cm, 20–40 cm, 40–60 cm, 60–80 cm, and 80–100 cm were monitored using a PICO–BT TDR instrument (IMKO, Ettlingen, Germany) at a distance of 30 cm from a *Lycium barbarum* trunk, with measurements taken approximately every five days. Meanwhile, additional measurements were taken before and after irrigation and rainfall, and soil samples were periodically collected using a soil auger, with calibration performed through the drying method.

#### 4.3.2. Plant Height and Stem Diameter Growth Amounts

Three *Lycium barbarum* trees with similar growth situations were selected in each plot area for tag marking, and a tape measure was used to measure the height from the base to the highest point of each tree, which is viewed as the plant height (cm) of the tree. Each *Lycium barbarum* tree was marked at a height of 5 cm from the ground surface, with an electronic digital caliper used to measure its stem diameter (mm) along the south–north and east–west directions individually. Measurements were taken at the end of each growth period, with the difference values between these two measurements regarded as the plant height and stem diameter growth amounts of *Lycium barbarum* during the growth period.

#### 4.3.3. Chlorophyll Content

Three pieces of complete and healthy *Lycium barbarum* leaves were randomly taken from each tree with treatment tag marking, and their chlorophyll contents (SPAD) were measured using a portable chlorophyll meter (SPAD–502 model, Konica Minolta, Tokyo, Japan), with their average values finally calculated.

#### 4.3.4. Photosynthetic Characteristics

Three pieces of complete and healthy *Lycium barbarum* leaves were randomly taken from each tree with treatment tag marking, with their daily variations in photosynthetic characteristics (net photosynthetic rate (*Pn*, μmol·m^−2^·s^−1^), transpiration rate (*Tr*, mmol·m^−2^·s^−1^), and intercellular CO_2_ concentration (*C_i_*, μmol·mol^−1^) measured over three consecutive days during its full bearing period using a photosynthesis meter (LI-6400 model, LI-COR, Lincoln, NE, USA). Measurements were arranged from 8:00 to 18:00 and taken every two hours each day, with their average values finally calculated.

The calculation formula for carboxylation efficiency (*CE*, mmol·m^−2^·s^−1^) is as follows:

(3) $$CE=P_{n}/C_{i}.$$

The calculation formula for leaf instantaneous water use efficiency (*LWUE*, μmol·mmol^−1^) is as follows:

(4) $$CE=P_{n}/C_{i}.$$

#### 4.3.5. Yield

No *Lycium barbarum* trees bore fruits in 2021. Therefore, the yields of all trees were not calculated in that year. The yields of dry *Lycium barbarum* fruits in 2022 were calculated using a method of cumulative yield of multiple harvests. After *Lycium barbarum* trees moved into their full bearing stage, three trees with consistent growth situations were selected and harvested in each plot area, with harvesting conducted every 7–9 days. During each harvest, picking, dewaxing, and drying were conducted based on a unit of a single *Lycium barbarum* tree, and the single-plant dried fruit yield under each treatment was measured using a weighing method and converted into a yield per unit area (kg·ha^−1^).

### 4.4. Data Analysis

A data statistical analysis was performed using Microsoft Excel 2016, and variance analysis and significance testing (*p* < 0.05) was conducted using IBM SPSS Statistics 26.0, with a diagram plotted using Origin 2021.

## 5. Conclusions

Plant height and stem diameter growth amounts of *Lycium barbarum* continuously increase with the increase in the irrigation amount, and increase first and then decrease with the increase in the nitrogen application amount, with W4N2–treated plants presenting maximum growth amounts of plant heights and stem diameters. Appropriate water and nitrogen supply is conducive to improving the leaf photosynthetic physiological characteristics of *Lycium barbarum* plants in a *Lycium barbarum* + Alfalfa system. W4N2–treated *Lycium barbarum* plants present the highest level of SPAD. Meanwhile, these plants present the highest levels of daily average *Pn*, *Tr*, and *CE*, which are 4.92–48.53%, 3.21–59.95%, and 10.13–129.52% higher than those of plants treated with other modes, respectively. W1N2–treated plants present the highest level of daily average IWUE (3.31 μmol·s^−1^). W4N2–treated *Lycium barbarum* plants present the highest dried-fruit yield (2623.07 kg·ha^−1^), while W3N2–treated plants present the highest net income (50,070 CNY·ha^−1^). Regression analysis shows that an irrigation amount range of 4367.33–4415.07 m^3^·ha^−1^ combined with a nitrogen application amount range of 339.80–367.35 kg·ha^−1^ constitutes an appropriate water and nitrogen supply control for increasing yields and profits of *Lycium barbarum* crops in the Yellow River Irrigation Area in Gansu, China and other similar ecological areas.

## Figures and Tables

**Table 1:** Effects of water and nitrogen regulation on yield and economic benefit of *Lycium barbarum.*

W	N	Irrigation Amount (m^3^·ha^−1^)	Yield (kg·ha^−1^)		Economic Benefits			
			Dry Weight	Fresh Weight	Total Income (×10^4^ CNY·ha^−1^)	Total Cost (×10^4^ CNY·ha^−1^)	Net Income (×10^4^ CNY·ha^−1^)	Input–Output Ratio
W1	N0	2475.09	1424.93 i	5099.49 h	5.13 i	2.86 g	2.27 f	1.79 h
	N1	2410.16	1566.56 h	5342.62 gh	5.64 h	2.84 g	2.80 e	1.99 ef
	N2	2540.60	1690.24 g	5878.4 g	6.08 g	2.92 g	3.16 d	2.08 de
	N3	2612.09	1566.14 h	5672.99 gh	5.64 h	2.75 g	2.89 de	2.05 de
W2	N0	3150.11	1873.35 f	6906.36 f	6.74 f	3.52 e	3.23 d	1.92 fg
	N1	2905.85	1986.88 f	7026.51 f	7.15 f	3.44 ef	3.71 c	2.08 de
	N2	3233.47	2117.5 e	7444.83 def	7.62 e	3.50 ef	4.13 b	2.18 bc
	N3	3324.47	1994.58 f	7116.63 ef	7.18 f	3.28 f	3.9 bc	2.19 bc
W3	N0	3825.13	1899.94 f	7245.93 ef	6.84 f	3.66 de	3.18 d	1.87 gh
	N1	3528.56	2247.62 d	8136.67 c	8.09 d	3.85 cd	4.24 b	2.1 cd
	N2	3926.39	2521.14 ab	8843.86 b	9.08 ab	4.01 bc	5.07 a	2.26 ab
	N3	4036.85	2339.56 cd	7984.34 cd	8.42 cd	3.6 e	4.82 a	2.34 a
W4	N0	4500.05	1902.05 f	7741.42 cde	6.85 f	3.85 cd	2.99 de	1.78 h
	N1	4151.24	2254.65 d	8804.01 b	8.12 d	4.11 b	4.01 bc	1.98 ef
	N2	4619.28	2623.07 a	10668.14 a	9.44 a	4.67 a	4.77 a	2.02 de
	N3	4749.22	2451.05 bc	8894.13 b	8.82 bc	3.95 bc	4.88 a	2.24 ab
F	W	–	293.403 **	211.238 **	293.403 **	234.409 **	161.587 **	21.775 **
	N	–	96.613 **	33.792 **	96.613 **	20.572 **	124.943 **	93.061 **
	W × N	–	7.697 **	4.926 **	7.697 **	4.934 **	7.179 **	3.152 **

Note: The data in the table are means. Urea—3.25 CNY·kg^−1^; superphosphate—1 CNY·kg^−1^; potassium chloride—4 CNY·kg^−1^; irrigation water and electricity—0.33 CNY·m^−3^. The labor fee is 150 CNY per person per day, the fresh wolfberry fruit picking fee is 3.5 CNY·kg^−1^, and the remaining cost of each processing is calculated according to the actual situation. In 2022, the average price of dried Chinese wolfberry was 36 CNY·kg^−1^. Different lowercase letters indicate a significant difference in yield and economic benefit under different water and nitrogen treatments (*p* < 0.05). W is water mode, N is nitrogen application level, and W × N is their interaction. ** indicates a very significant difference (*p* < 0.01).

**Table 2:** Experimental design.

Treatment	Irrigation Mode (% *θ_f_*)	Nitrogen Application Level (kg·ha^−1^)
W1N0	45–55 (Severe water deficit)	0
W1N1		150
W1N2		300
W1N3		450
W2N0	55–65 (Moderate water deficit)	0
W2N1		150
W2N2		300
W2N3		450
W3N0	65–75 (Mild water deficit)	0
W3N1		150
W3N2		300
W3N3		450
W4N0	75–85 (sufficient irrigation)	0
W4N1		150
W4N2		300
W4N3		450

Note: *θ_f_* is the field capacity, the lower limit of irrigation before “–”, and the upper limit after “–”; The planned depth of the wet layer is 60 cm.

## Data Availability

All data supporting this study are included in the article.
